# Shifting Leadership Paradigms in Healthcare and Public Health

**DOI:** 10.3389/phrs.2025.1608588

**Published:** 2025-12-08

**Authors:** Behrooz Behbod, Katarzyna Czabanowska

**Affiliations:** 1 The Entrepreneur’s Doctor, Cardiff, United Kingdom; 2 Department of International Health, Care and Public Health Research Institute (CAPHRI), FHML, WHO CC for Public Health Leadership and Workforce Development, Maastricht University, Maastricht, Netherlands; 3 Department of Health Policy Management, Institute of Public Health, Faculty of Health Sciences, Jagiellonian University, Krakow, Poland

**Keywords:** leadership, burnout, wellbeing, public health leadership, leadership capability, natural leadership

## Introduction

Healthcare and public health systems are navigating extraordinary complexity. The pressures of workforce shortages, documentation burdens, and widening health disparities have exposed deep structural vulnerabilities.

Burnout among health professionals has reached critical levels. A 2024 meta-analysis found that 42% of public health workers worldwide reported burnout during the pandemic, compared to 35% pre-pandemic [1]. These figures reflect trends in the public health workforce and should not be conflated with clinical settings, where burnout rates vary by role and geography. Burnout contributes to serious health outcomes including depression, cardiovascular disease, and insomnia [2].

This highlights the urgency of exploring new approaches to leadership and wellbeing. While operational reforms are essential, they are not sufficient. This commentary explores a different kind of leadership; one that begins within.

Rather than focusing solely on external strategies or frameworks, this approach invites leaders to reconnect with their innate capacity for clarity, resilience, and insight. It is grounded in the understanding that how we experience the world is shaped from within. When leaders operate from a settled mind and a clear perspective, they are better equipped to respond to challenges with wisdom and presence.

## Leadership as an Effect Modifier

In environments marked by volatility, uncertainty, complexity, and ambiguity, conditions that challenge stability and clarity, leadership must do more than manage; it must stabilise and catalyse. This understanding of human experience offers a unique contribution: it acts as a buffer against harmful occupational exposures such as stress, overwhelm, and burnout, and as a catalyst for the qualities leadership demands; clarity, adaptability, and grounded decision-making.

This is not a rejection of system-level interventions. On the contrary, it complements them. When leaders are internally resourced, they are more capable of engaging with structural reforms, navigating complexity, and supporting their teams effectively.

## Evidence and Emerging Research

While this understanding is gaining traction, the empirical evidence base is still emerging. The principles underlying the inside-out paradigm are explored in an article by Kessel and colleagues in the *Journal of Public Mental Health* [3]. Two recent studies have begun to examine its impact on mental wellbeing, resilience, and the ability to navigate challenges among children and adolescents [4, 5]. These early findings suggest potential benefits worth investigating further in adult and professional populations.

This commentary does not claim definitive outcomes. Instead, it makes the case for building a robust evidence base to evaluate the efficacy and effectiveness of this approach in leadership contexts.

## From Insight to Action

When leadership arises from within, grounded in clarity, presence, and a settled mind, it does not need to be forced or manufactured. It flows naturally. Leaders who operate from this space are not reacting to pressure; they are responding with wisdom. Their actions are not driven by fear or control, but by insight and alignment.

Here’s how this might look in practice:

### Health Workforce

A manager walks into a team meeting not with a list of solutions, but with a quiet mind and an open heart. They listen, not to fix, but to understand. In that space, new ideas emerge. They recognise the need for breathing room and advocate for temporary staffing support. They notice the burden of redundant reporting and initiate automation. They sense the value of pausing and create monthly “stop-the-line” fora, a concept adapted from high-reliability industries like aviation and manufacturing, where any team member is empowered to pause operations when they notice a problem or risk, so it can be addressed immediately before it escalates. In a healthcare or public health context, a “stop-the-line” forum refers to a regular, protected space where staff can raise concerns, share observations, or reflect on challenges without fear of reprisal. It’s not just a meeting; it’s a psychologically safe environment where the implicit message is: *“If something does not feel right, let’s pause and talk about it.”* These actions do not come from strategy; they come from clarity. The impact? A team that feels seen, supported, and reconnected with purpose.

### Healthcare Organisation

A physician, overwhelmed by the pace and paperwork, takes a moment to reconnect with why they chose medicine. In that pause, they realise that presence, not productivity, is what heals. They pilot a documentation-relief strategy, not to tick boxes, but to reclaim space for connection. With scribes and templates easing the load, they find themselves more available, more human. Patients feel it. So does the physician. After-hours charting drops. Visit overruns shrink. The work feels meaningful again.

### Community Engagement

A public health leader stops trying to solve problems from a distance. Instead, they sit with community members, not as an expert, but as a fellow human. In that shared space, stories unfold. Insights arise. Together, they co-create solutions that reflect lived experience. The leader does not push an agenda; they follow the thread of what makes sense. Protected collaboration time is introduced. Engagement metrics rise. But more importantly, trust is built.

### Planetary Health

A climate-focused team, facing overwhelming data and urgency, chooses to slow down. They gather not just scientists, but artists, educators, and citizens. They do not start with answers; they start with curiosity. In that openness, creativity flourishes. Campaigns become conversations. Policy becomes possibility. The shift is not just in outcomes; it’s in energy. People feel invited, not instructed, and they respond.

In each of these examples, leadership is not a performance, it’s a presence. It’s not about doing more, it’s about seeing more clearly. When leaders lead from this space, their actions are not only more effective, but they’re also more humane.

## Comparative Positioning

This potentially transformative approach shares values with compassionate, collective, and systems leadership, such as transparency, ethical grounding, and relational care. However, it differs in its *locus of change*. Rather than starting with external structures or team dynamics, it begins with the individual’s capacity for insight and presence.

In contexts requiring rapid operational change, systems leadership may offer more actionable levers. In relational or high-stakes environments, collective and compassionate models may be more resonant. Leading from within adds depth by stabilising the leader’s internal state, potentially enhancing the effectiveness of any external strategy.

## Equity and Power

Leadership from within must be practiced with care. There is a risk that “being true to oneself” can rationalise bias or blur boundaries. In hierarchical systems, this may cause harm. Safeguards are essential: fair-process decision-making, equity checks, protected dissent channels, and external accountability mechanisms ensure that clarity and authenticity serve the collective good.

## Scope and Audience

This commentary addresses both public-health agencies and healthcare delivery organisations. Sector-specific examples and metrics are presented separately to respect the unique challenges and contexts of each.

### Conclusion

Leadership is not merely a set of skills we acquire; it emerges when we uncover the clarity and wisdom already present within us. When leaders reconnect with their innate clarity and pair it with system-level commitments, they unlock potential in themselves, their teams, and the systems they serve. This approach does not replace structural reform; it enhances it. It offers a stabilising force in turbulent times and a source of creativity and resilience in the face of complexity.

This is an invitation to explore further, to build the evidence base, and to consider how leadership might evolve when we begin from within.
